# Access to HIV prevention and care for HIV-exposed and HIV-infected children: a qualitative study in rural and urban Mozambique

**DOI:** 10.1186/1471-2458-14-1240

**Published:** 2014-12-03

**Authors:** Caroline De Schacht, Carlota Lucas, Catarina Mboa, Michelle Gill, Eugenia Macasse, Stélio A Dimande, Emily A Bobrow, Laura Guay

**Affiliations:** Elizabeth Glaser Pediatric AIDS Foundation, Avenida Kwame Nkrumah 417, Maputo, Mozambique; Elizabeth Glaser Pediatric AIDS Foundation, 1140 Connecticut Ave NW, Suite 200, Washington, DC, 20036 USA; Central Hospital Maputo, Mozambique, Av. Eduardo Mondlane/Av. Agostinho Neto, Maputo, Mozambique; Provincial Health Directorate, Matola, Maputo Province, Mozambique, Praça do Município 111.29, Matola, Mozambique; Department of Epidemiology and Biostatistics, The George Washington University, Milken Institute School of Public Health, 950 New Hampshire Ave NW, 5th floor, Washington, DC, 20052 USA

**Keywords:** Prevention of mother-to-child transmission, Barriers, Motivators, Pediatric HIV, Mozambique, HIV-exposed children, Infant HIV testing, Early infant diagnosis

## Abstract

**Background:**

Follow-up of HIV-exposed children for the delivery of prevention of mother-to-child transmission services and for early diagnosis and treatment of HIV infection is critical to their survival. Despite efforts, uptake of postnatal care for these children remains low in many sub-Saharan African countries.

**Methods:**

A qualitative study was conducted in three provinces in Mozambique to identify motivators and barriers to improve uptake of and retention in HIV prevention, care and treatment services for HIV-exposed and HIV-infected children. Participant recommendations were also gathered. Individual interviews (n = 79) and focus group discussions (n = 32) were conducted with parents/caregivers, grandmothers, community leaders and health care workers. Using a socioecological framework, the main themes identified were organized into multiple spheres of influence, specifically at the individual, interpersonal, institutional, community and policy levels.

**Results:**

Study participants reported factors such as seeking care outside of the conventional health system and disbelief in test results as barriers to use of HIV services. Other key barriers included fear of disclosure at the interpersonal level and poor patient flow and long waiting time at the institutional level. Key facilitators for accessing care included having hope for children’s future, symptomatic illness in children, and the belief that health facilities were the appropriate places to get care.

**Conclusions:**

The results suggest that individual-level factors are critical drivers that influence the health-seeking behavior of caregivers of HIV-exposed and HIV-infected children in Mozambique. Noted strategies are to provide more information and awareness on the benefits of early pediatric testing and treatment with positive messages that incorporate success stories, to reach more pregnant women and mother-child pairs postpartum, and to provide counseling during tracing visits. Increasing uptake and retention may be achieved by improving patient flow at the institutional level at health facilities, by addressing concerns with family decision makers, and by working with community leaders to support the uptake of services for HIV-exposed children for essential preventive care.

## Background

By the end of 2012, an estimated 35 million people were living with HIV globally, including 3.3 million children less than 15 years old. The number of HIV-positive children receiving antiretroviral therapy (ART) has increased; however, only 34% of the children eligible for treatment globally are accessing ART, compared to 65% for adult coverage [1].

Engagement in care for prevention of HIV infection with early diagnosis of HIV-exposed children and for treatment initiation for HIV-positive children is critical to their survival. Early infant death was reduced by 76% in infants who began treatment at diagnosis compared with those whose therapy was deferred until immunologic decline, with the largest mortality differences found among the youngest infants (6–25 weeks) [2].

In 2010, the World Health Organization identified some key challenges to providing ART to HIV-positive children, which included: limited screening for HIV; lack of affordable, simple diagnostic technologies; insufficient human capacity; insufficient advocacy; limited experience in HIV care; and lack of pediatric antiretroviral formulations [3].

Despite the efforts in simplifying drug regimens and inclusion criteria to improve access to ART care, many challenges in identifying and following HIV exposed and infected children still remain [4]. In Botswana for example, 71% of the infants have been tested for HIV through early infant diagnosis between 2005 and 2012. Of the HIV positive infants, only 41% were alive and on treatment at the time of the study [5]. In Ivory Coast, 20% of the children diagnosed with HIV were lost to follow up even before treatment was initiated [6]. Another study done in three sub-Saharan countries also demonstrated missed opportunities in linkages between the PMTCT and ART programs [7]. These studies however did not study any underlying influencing factors.

Data on barriers to ART access for children are limited; however, the main challenges reported in studies done in Southern Africa include individual-level (knowledge, fear of women’s and child’s HIV status, stigma) and institutional-level factors (queues, attitude of health care workers, inconsistent referral) [8–10]. A study in South Africa showed that when ART and prevention of mother-to-child transmission (PMTCT) programs are not integrated or linked in some way there may be missed opportunities for the continuous care for HIV-positive mothers and their HIV-exposed children [11].

Mozambique is highly affected by HIV with a prevalence of 16% among pregnant women in 2011 [12]. The 2012 Global AIDS Response Progress Report for Mozambique reported that one of the challenges of the PMTCT program is increasing the coverage of follow up for HIV-exposed infants and children. In 2010, an estimated 64% of the HIV-exposed infants in Mozambique received antiretroviral prophylaxis post-delivery [13].

The Elizabeth Glaser Pediatric AIDS Foundation has supported the national PMTCT and ART programs in four provinces in Mozambique since 2004. This includes expansion of services for early infant diagnosis of HIV, provision of pediatric ART in district-level health facilities, and integrated HIV care and treatment for pregnant and postpartum women within maternal and child health clinics. Despite this, only approximately 27,000 HIV-positive children were initiated on treatment by the end of 2012 [1], representing 27% of children in need of ART [1]. A study in the central region of the country showed that only 25% of the infants born to HIV positive women returned for early infant diagnosis [14]. Uptake of and retention in HIV services for the first two years of life for HIV exposed children and for the life time of HIV infected children involves the complex interplay between a parent/caregiver’s own health status and medical needs and the needs of one or more exposed/infected children. This is also affected by multiple spheres of influence on the caregiver’s beliefs and health seeking behaviors. This qualitative study sought to explore multilevel barriers to and motivators for accessing HIV prevention, care and treatment services for children from the different perspectives of caregivers, health care workers and influential people using a socioecological framework. Study results, including recommendations from participants, will contribute to addressing the gaps in HIV prevention, care and treatment programs in Mozambique and elsewhere to minimize new infections in children and maximize survival of those infected.

## Methods

### Study design and study sites

A qualitative cross-sectional study was conducted in three provinces of Mozambique. The study included key informant interviews with parents/caregivers, health care workers (HCW), and community leaders; and focus group discussions (FGD) with parents/caregivers, grandmothers, and community leaders. FGDs were used as the sole data collection method for grandmothers in order to provide a more familiar setting where they could interact with their peers and thus obtain richer data through the interactions among the women. HCWs participated only in key informant interviews due to their varied backgrounds and positions within the facilities.

Government health facilities and the surrounding catchment areas representing rural, semi-urban and urban locations and different facility levels were purposively selected from Cabo Delgado Province in the north (Macomia Health Center and Montepuez Rural Hospital), Maputo Province in the south (Magude Health Center and Marracuene Health Center) and the Central Hospital located in Maputo City. Maputo Province was selected due to the high HIV burden in the south, and Cabo Delgado Province was selected due to challenges in accessing HIV care for children in the north. The Central Hospital was included to represent an urban setting. At the time of the study, Option A was used as a national guideline for PMTCT care with follow-up of HIV-exposed children was done at district-level, including a monthly check-up, provision of prophylaxis and sample collection for early infant diagnosis and rapid testing of HIV. HIV-infected children were followed with a monthly clinic visit at the district health facility and were offered prophylaxis and antiretroviral therapy following the national guidelines, including antiretroviral treatment for all HIV-positive children less than one year of age.

### Study population

The study included four distinct populations in each province. Parents and other caregivers (all referred to as caregivers) were defined as adults having primary care-giving responsibility for at least one HIV-positive or HIV-exposed child 0–14 years old at the time of the study. Caregivers were either attending maternal and child health services in the study health facilities or had defaulted from care, defined as missing the clinic visit for at least one month and were identified in the community by lay counselors. Grandmothers were included in the study population because of their influential role as the mother-in-law in families within the Mozambican context and were defined as women with at least one grandchild who was 0–14 years old at the time of the study, regardless of HIV exposure status. HCW included maternal and child health nurses and lay counselors who were involved in PMTCT and pediatric HIV care services. Lay counselors provided psychosocial support to families affected by HIV in study facilities and were involved in active follow-up of HIV-exposed and HIV-positive children. Maternal and child health nurses and lay counselors were combined for the purposes of analysis. Community leaders included those who played an influential role in the communities surrounding the study facilities.

### Recruitment procedures

Caregivers in study facilities were identified and introduced to the study by HCW. Those willing to consider study participation were referred to trained study staff members. Caregivers who defaulted were identified by HCW through the existing active tracing systems utilized by the health facilities in the catchment area. Those who had missed clinic visits were first approached by HCW conducting routine home visits where they were counseled on adherence to HIV prevention and treatment, encouraged to return to the facility for care and briefly informed about the study. Interested caregivers were referred to study staff, who screened them for eligibility and conducted interviews after obtaining informed consent, either at the time of the initial introduction to the study or at another time and place, depending on the caregivers’ preference. Following initial introduction to the administrative authorities in facility catchment areas, the study team identified and recruited community leaders. Community leaders identified grandmothers in the communities surrounding study facilities and invited them to a location where study staff was present to conduct a FGD. HCW were identified by facility administrators. Once referred to the study team, all potential participants received further information about the study and if interested, underwent the informed consent process. Only individuals who were 18 years of age or older and provided written informed consent were included in the study.

### Data collection and analysis

Data were collected between August 2011 and January 2012 by trained study staff familiar with the local context. Guides contained questions on demographics and open-ended questions on knowledge, attitudes, behavior, perceptions, and recommendations for contributing factors related to improving access to and follow-up for HIV-exposed and HIV-infected children. FGD were guided by vignettes depicting hypothetical scenarios experienced by caregivers of HIV-exposed and HIV-positive children. FGD and interviews were conducted in Portuguese or a local language. Audio recordings and notes from all the FGD and interviews were translated into Portuguese and transcribed into Microsoft Word. Demographic information was entered into CSPro Version 4.1 (Census Bureau, Washington, USA).

The transcripts were manually coded for content by two researchers working independently. Codes were developed inductively and the main themes were identified. The results of the two researchers were compared to assess inter-rater reliability; consensus was reached in areas of disagreement. MAXqda Version 10 (Verbi GmbH, Berlin, Germany) was used for analysis of the qualitative data. Participant demographic data were analyzed using the statistics software package Stata Version 11.0/SE (StataCorp, Texas, USA) and were summarized using descriptive statistics only.

The study was approved by the National Health Bioethics Committee of Mozambique and the George Washington University Institutional Review Board.

### Conceptual framework

**Figure 1:**
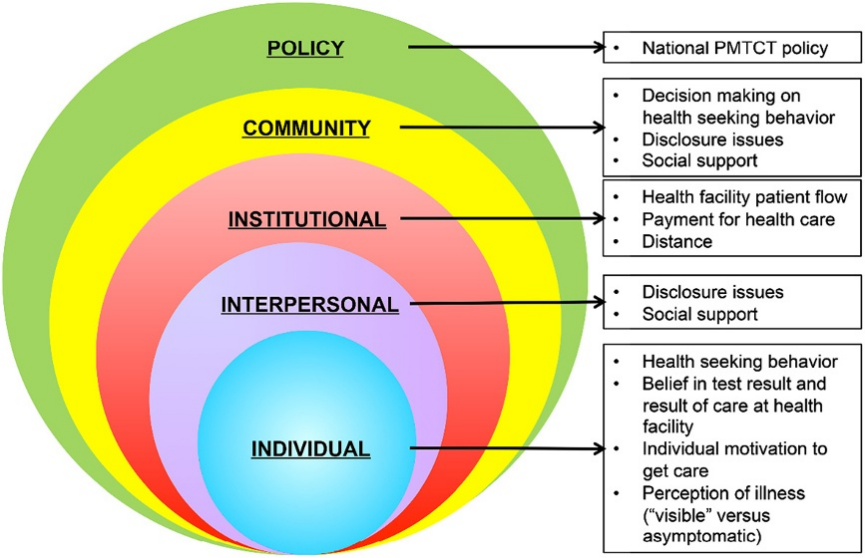
**The Conceptual Framework reflecting the five levels of influence on access to HIV prevention and care for HIV-exposed and HIV-infected children in Mozambique.**

A conceptual framework was elaborated and adapted from the socioecological framework developed by McLeroy et al. [15], organizing the qualitative results into five levels of influence: individual, interpersonal, institutional, community and policy. Figure 1 displays the factors included in the framework. Individual-level factors were defined as a person’s knowledge, perceptions and behavior specifically focused on health-seeking behaviors, acceptance of HIV test results, motivators to access of care, and perceptions of illness as being “visible” versus asymptomatic. Interpersonal-level factors included issues of disclosure within the family, social support within the family, and general social support. Institutional-level factors centered on the health facilities available in communities with important determinants including patient flow, payment for health care, and distance needed to travel to access care. Community-level factors included influences on decision making to seek care, disclosure beyond the family, and wider issues of social support. At the policy level, the highlighted determinant was defined as the content and implementation of the National PMTCT policy which dictates how facilities provide care for HIV-exposed and HIV-infected children.

## Results

### Study participants characteristics

A total of 79 individuals were interviewed: 23 caregivers (adherent to care) recruited from study health facilities, 20 caregivers (lost to care) recruited from communities, 16 HCW and 20 community leaders. In addition, 31 FGD were conducted with 48 caregivers recruited from study health facilities (8 FGD), 59 grandmothers (8 FGD), 28 healthcare professionals (5 FGD), and 76 community leaders (10 FGD).

The demographic characteristics of the study populations are presented in Table 1. Caregivers recruited at the facility were predominantly female (96%) versus those recruited in the community (75%). More caregivers recruited at the facility were married or lived with their male partner than those in the community (76% versus 45%). The community leaders were predominately male (75%) with a median age of 58 years (IQR 48–68). Characteristics of the caregivers enrolled from the facilities and those identified in the community and their children are included in Table 2. The caregiver was the biologic parent of the HIV-exposed or infected child in 89% of the cases. Of the 91 children of caregivers, 43% (N = 39) were HIV-exposed and 57% (N = 51) were HIV-infected (one missing). The median age of HIV-infected children is higher in comparison to that of HIV-exposed children (6 years (IQR 1–8) versus 1 year (IQR 0–1); p = 0.0001). Children from caregivers recruited in the community were older than the ones recruited in the health facility (median age 3.5 years (IQR 1.5-9) versus 1 year (IQR 0–5); p = 0.002). None of the caregivers recruited in the community had ever participated in a support group, in contrast to 33% of caregivers recruited at study health facilities.

**Table 1:** **Demographic characteristics of the study populations (Interviews and Focus Group Discussion)**

	Caregivers (facility-recruited) n (%) – median (IQR)	Caregivers (community-recruited) n (%) – median (IQR)	Grandmothers n (%) – median (IQR)	Community leaders n (%) – median (IQR)	Health care professionals n (%) – median (IQR)	TOTAL n (%) – median (IQR)
**Number of participants**	**71**	**20**	**59**	**96**	**44**	**290**
Sex*						
Female	67 (96)	15 (75)	59 (100)	24 (25)	27 (61)	192 (67)
**Age (years)**	30 (25–38)	32 (24–42)	53 (43–60)	58 (48–68)	29 (26–34)	42 (30–58)
Marital status**						
Single	7 (10)	2 (10)	4 (7)	5 (5)	10 (23)	28 (10)
Married/living together	53 (76)	9 (45)	35 (59)	82 (86)	34 (77)	213 (74)
Separated/divorced	5 (7)	4 (20)	6 (10)	3 (3)	0	18 (6)
Other	5 (7)	5 (25)	14 (24)	6 (6)	0	30 (10)
Education*						
No education	20 (29)	4 (20)	34 (58)	21 (22)	0	79 (27)
Basic education (grade 6)	39 (56)	12 (60)	22 (37)	67 (71)	5 (11)	145 (50)
Medium level (grade 10)	10 (14)	3 (15)	2 (3)	7 (7)	25 (57)	47 (16)
Pre-university (grade 12)	1 (1)	0	1 (2)	0	12 (27)	14 (5)
University or above	0	1 (5)	0	0	2 (5)	3 (1)
Employment status*						
Yes, full time	5 (7)	4 (20)	3 (5)	13 (14)	36 (82)	61 (21)
Yes, part time	15 (21)	5 (25)	2 (3)	11 (11)	7 (16)	40 (14)
No	50 (71)	11 (55)	54 (92)	71 (75)	1 (2)	187 (65)

*Two missing responses; **1 missing response.

**Table 2:** **Characteristics of the caregivers and their children**

	Caregivers from facilities n (%) – median (IQR)	Caregivers from communities n (%) – median (IQR)	TOTAL n (%) – median (IQR)
**Number of participants**	**71**	**20**	**91**
Serostatus of child*			
HIV-exposed	34 (49)	5 (25)	39 (43)
HIV-infected	36 (51)	15 (75)	51 (57)
**Age of child (years)**	1 (0–5)	3.5 (1.5-9)	1 (1–6)
Sex of child*			
Male	38 (54)	10 (50)	48 (53)
Female	31 (44)	10 (50)	41 (46)
Twin (Male/Female)	1 (1)	0	1 (1)
Relationship to child*			
Parent	62 (89)	18 (90)	80 (89)
Legal Guardian	2 (3)	0	2 (2)
Informal Guardian	6 (9)	2 (10)	8 (9)
Number of children in household*			
0	0	1 (5)	1 (1)
1	14 (20)	2 (10)	16 (18)
2	13 (19)	7 (35)	20 (22)
3	17 (24)	7 (35)	24 (27)
4	13 (19)	3 (15)	16 (18)
5	8 (11)	0	8 (9)
>5	5 (7)	0	5 (5)
Live within facility catchment area*			
Yes	10 (14)	6 (30)	16 (18)
Usually attend this facility*			
Yes	68 (96)	17 (85)	85 (93)
Attend any support group**			
Yes, in this facility	22 (32)	0	22 (25)
Yes, in other facility	1 (1)	0	1 (1)
No	46 (67)	19 (100)	65 (74)

*One missing response; **three missing responses.

### Qualitative results

Qualitative results were organized into barriers, motivators and recommendations in the main levels of influence. Regional differences are included in the results, but no contrasting findings were found by child HIV infection status. Table 3 summarizes the various barriers, motivators, and recommendations identified by study participants.

**Table 3:** **Summary of barriers, motivators and recommendations about accessing HIV prevention and care for children**

	Barriers	Motivators	Recommendations
Individual*	• Seeking alternative care for illness	• Health facility is the appropriate place to receive care	• Provide more information and awareness in the community about HIV
	• Disbelief in HIV test results (mom and/or infant)	• Hope for future of the child	
	• Lack of motivation to attend facility care	• Having symptoms (visible illness)	
	• Fatalistic beliefs about HIV/AIDS		
**Interpersonal**	• Lack of social support from partners and family	• Family being supportive regarding HIV care	• *No specific recommendations were given by the participants on this level*
	• Fear of disclosure to partners	• Family decision-maker in favor of care at the health facility	
	• Generation conflict (Southern region)		
**Institutional**	• Long waiting times for services (patient flow)	• *No specific motivators were mentioned by the participants on this level*	• Improvements to patient flow
	• Distance to health facility		• Organizing outreach clinics and home-based care for HIV counseling, testing, and follow-up
			• Including messages of hope in education to the community
	• Money for transport to health facility		
**Community**	• Lack of social support from community	• Social support from community members	• Support by community members (for example home visits, awareness session given by members)
	• Fear of disclosure to those in the community		• Support by community in referring to health facilities (for example active referral by leaders)
			• Community support groups with refreshments provided
			• Financial incentives for counselors of the support groups
**Policy**	Non-integrated services	• Services free of charge	• Integrate nutritional support services with HIV services

*Most frequently mentioned responses were at the individual level.

1. *Individual-level factors*
  In all groups and provinces, participants frequently reported seeking alternative care before attending services at health facilities. Alternative care was explained by respondents as delaying, interfering, or replacing HIV testing, care and treatment from a health facility. Respondents explained that some diseases have spiritual causes that cannot be treated in health facilities and should be dealt with using traditional rituals before medical personnel can address the physical ailment.
  …*“So, in relation to AIDS; what they do is to push away the bad spirits so that the person becomes clean, and then send him to the doctor so he can see him because if my organism (body) is not pure (from bad spirits), and I go to the doctor first, he will simply say that I am seronegative, because he cannot see because the bad spirits from our grandparents are locked up…” [Focus Group Discussion, Community leader, South]*
  Some caregivers recruited from health facilities in the northern and southern provinces who acknowledged that they seek alternative care for them or their children, said that they do so if their health does not improve through facility-based care.
  *“I have gone to traditional healers as well, but I start coming here at the hospital and if there is no improvement I take him (the child) to the traditional healer, but if their things are also not good, I end up staying hopeless at home.” [Focus Group Discussion, Caregiver at facility, North]*
  Caregivers’ disbelief in their own, their partners’ or child’s positive HIV test result, or being asymptomatic was associated with non-acceptance of an HIV diagnosis, resulting in not seeking HIV care, particularly in Maputo Province and City. All groups mentioned their distrust of HIV testing technologies as an impediment to seeking services for themselves and their children.
  *“I believe in the test result of my child but not mine. If I am HIV positive, why haven’t I started treatment yet?” [Individual Interview, Caregiver in Community, South]*
  Participants from the southern study sites report the lack of courage to face reality about their positive test result as a barrier to care. Other barriers included lack of caregiver motivation and interest to seek chronic care for the child. Some respondents believe that HIV is a fatal disease and thus do not feel the need to seek care for the children, as they believe the child will die anyway.
  All groups from all provinces mention that having a visible illness or wanting to know about the health status of their children was one of the most important motivators to accessing care at health facilities.
  *“..I am coming back because the child is not well, the body is warming up a lot and he is not eating well” [Individual interview, Caregiver in community, South]*
  Mainly caregivers, both recruited at health facilities and in communities, reported that seeing people living with HIV who receive facility-based care with improved health and longevity gave caregivers hope for their infected or exposed children.
  *“..what I want for my child is that he is healthy; just as they took care of me until I am as I am now, I also want that my child has a future.” [Focus Group Discussion, Caregiver at facility, South]*
  Some participants, particularly caregivers recruited from health facilities and community leaders, felt that facilities were the most appropriate place for HIV care. In some cases, traditional healers act as motivators by referring their patients to health facilities.
2. *Interpersonal-level factors*
  The presence of family support, for example helping to remind the main caregiver about follow-up visits, was discussed as facilitating access to HIV testing, care and treatment. Not having any emotional or social support hindered access. Fear of disclosure within the family as a barrier to engaging in HIV services was mentioned most frequently in the three southern study sites. Caregivers who took their children to health facilities risk inadvertent disclosure of their children’s status or their own, which may result in abandonment by women’s partners or other negative implications.
  *“…now she hides maybe because if the husband finds out he can send her away, because if he is diagnosed from you as woman, this is heavy. The disease is easier to handle with her when it starts hitting the husband as nobody will talk. But if by coincidence the disease comes to you as woman, these are big problems and you will have to take your stuff and go back to your parents’ home, so this is what scares women…” [Focus Group Discussion, Community Leader, South]*
  Caregivers reported that when they do not have sufficient social support, they tend to abandon care at health facilities. Decisions makers in the family are highly influential providers of social support. In Mozambique these are predominantly the male head of the household who tends to be the father of the child, or the grandmother (in the north this is the mother of the mother and in the south this is the mother of the father) in the absence of the man. Grandmothers, HCW and caregivers from communities said that caregivers are often obliged to follow instructions given by traditional healers for fear of being abandoned by their partners who oppose and mistrust conventional medicine. In the southern study sites, grandmothers described relationship conflict with their daughters-in-law as a barrier to seek care for children at the health facility. They explained that their in-laws did not discuss their HIV status and their need for care and preferred to avoid health facilities, which hinders grandmothers’ ability to provide support.
  Respondents from all groups and all provinces said that when the caregivers felt supported by their family, they were more willing to seek HIV care at health facilities. All groups except caregivers recruited at facilities reported that in cases where the decision-maker in the family was in favor of getting care at the health facility, caregivers were more open to and likely to visit the health facility for care.
3. *Institutional-level factors*
  Participants discussed service delivery at health facilities as playing a vital role in caregivers’ health-seeking behavior. Factors that were associated with reduced retention in care and treatment at facilities included long waiting times, often due to an insufficient number of providers, a separate process for drug pick-ups at the pharmacy that identifies patients as HIV-positive, and the need for multiple clinic visits often over a long duration of time to receive test results and care. These barriers were mentioned by all groups in all provinces.
  *“… They did blood tests of the child and said the blood will be taken to Pemba… three months passed, we always asked and they said the result has not returned yet… the fourth time they told me that my child does not have the disease, only that I have to go to the doctor; I went but I was told that the doctor does not work today and I should come back on Monday… I came back, but they did not work and they told me to come the next Monday. I came on Monday. They said to come back the next Monday…and I gave up to going to the hospital …” [Individual Interview, Caregiver at facility, North]*
  Transportation was also mentioned as a barrier with long distances to the facility as a problem in the north and lack of money to pay for transport in the south. Caregivers recruited from the community from all provinces, but especially from the southern province, reported to not have time to go to health facilities. In general, very few motivators were mentioned.
4. *Community-level factors*
  Lack of social support from community members (external to children’s families) such as neighbors, influential people in the community, and fear of disclosure beyond the family were the main barriers to accessing care. Psychosocial support mainly through community-based support groups was mentioned frequently in all provinces to be a motivator to seek HIV care. The important role of the community in making referrals to health facilities was highlighted. HCW pointed out that treatment services could be more effective if carried out through support group meetings, but additional incentives, such as refreshments during the group meeting and financial incentives for the counselors, are needed for groups to remain active.
  *“What happens in the community when we know that child x is sick, we usually support the parents and go and counsel them to get the child to the hospital and try to cure the disease; and we, women, go to visit them, specifically women from the “OMM” (Mozambique Women’s Organization) to see how the person is doing, if he/she goes to the hospital and takes the medication.. but mainly we incentivize them to go to the hospital. The thing of making fun of people doesn’t happen anymore. This is what we do in our community.” [Focus Group Discussion, Grandmothers, South]*
5. *Policy-level factors*
  The principal barrier at this level was related to the decentralization of pediatric HIV services in Maputo City, as part of the national policy. In this 2009 policy change, patients who previously attended the specialized pediatric clinic at Maputo Central Hospital were transferred to lower-level health units. The fact that services were no longer in a specialized clinic was seen as a barrier to seek care in the decentralized facilities. The provision of free services in the national health services was reported as an important motivator.
  *“our patients for example were used to be attended very quickly, now that they go to the others (meaning peripheral health facilities), there, they do not feel very good… and it takes a long time to be attended; others end up giving up because of those delays.. do you see?” [Individual Interview, Health Care Worker, Maputo City]*
6. *Participant recommendations*
  At the institutional level, participants offered different suggestions for improving health service delivery, with regional variations. In the southern province, participants recommended increasing the number of staff at health facilities to avoid the long waiting times that discouraged people from going for consultation. Other recommendations included facility-level improvements to patient flow in order to reduce long queues and the integration of HIV services in maternal and child health care. To improve access, recommendations included organizing outreach clinics and home-based care for HIV counseling, testing, and follow-up for HIV-exposed or infected children. Respondents appealed for better coordination between health facilities and communities. According to participants, information and communication materials concerning HIV should include more messages of hope for those infected.
  *“What could be improved mainly in the messages is that in principle it should be said that this disease has no cure, no cure, no cure, but it turns out that many people who take treatment are healthy and happy. Then they should disclose enough information that says let us go and do the treatment so that we can be healthy, and organize our lives. The message should be unique and not messages like, if you have this disease you will not live, you will not live, while there are people that are living with this disease.” [Individual interview, Community leader, South]*
  At the policy level, from respondents in Maputo City, recommendations were related to the 2009 policy change for the decentralization of ART services. Caregivers felt that adherence would be improved by reinstituting specialized clinics. Nutritional support, especially for those with infants beginning complementary feeding, is seen as a method to improve adherence to HIV services, and was mentioned frequently in all regions.
  *“…we all appeal that they (the ministry) let the children be taken care of here (in the reference center), well, we – who are adults can be transferred, but it is not worth to do with children; in the health facilities there is no good service and the children will die…” [Focus Group Discussion, Caregivers at Health Facility, Maputo City]*

## Discussion

This qualitative study conducted in three provinces in Mozambique revealed several barriers and motivators to improving access of and follow-up for HIV exposed and HIV infected children. The main barriers were the practice of seeking traditional medicine as a replacement for or a delay to care at a health facility, the disbelief in HIV test results, and the fear of disclosure of women’s or child’s test result mainly to partner and family. The prominent motivating factor to take children to facilities was having visible symptoms of an illness. Participants attributed an important role to traditional healers in diagnosing and treating diseases and reported seeking their services in place of, or prior to, facility services. The provision of traditional medicines to prevent and cure illnesses and those deemed to have spiritual causes is common, especially in the south. A study looking at barriers to exclusive breastfeeding in three regions in Mozambique showed that people go to traditional healers for medical care for their infants [16]. Giving “medicine of the little pot” (“*panelinha*”) to cure or prevent “moon disease”, which is said to cause mental retardation and madness, is common practice in the south [17]. Child illness in parts of the north is seen as a supernatural condition that can only be treated by the traditional healer. For example, diarrhea is believed to be related to sexual activity during the breastfeeding period, or by not following certain traditional ceremonies [18].

The belief that HIV is a fatal disease with no cure or treatment is a barrier to accessing care reported mainly by participants from the north. HIV is a fatal disease with no cure, and most people in Mozambique witnessed this among their HIV infected family and friends prior to the widespread availability of ART. In addition, this was emphasized as a tactic to promote safer behaviors in early prevention messages. However, more recent knowledge, understanding, and experience in seeing the benefits of treatment for HIV may not be as widespread and therefore should be addressed. In a national survey on HIV, only 27% of men and 34% of women in the north demonstrated comprehensive knowledge on HIV [19]. A low literacy level was seen to be related to lack of knowledge on HIV in central Mozambique [20]. The population density in the northern province is low, with long distances between villages and between villages and facilities, which may make it more difficult to disseminate health information, including HIV-related information. There may be nuances to communicate about the frequency and duration of taking ART for treatment and prevention, but the core issue, which makes this belief a barrier, is that people will not agree to seek HIV care at a health facility if they perceive that nothing can be done to improve their health or final outcome.

Being asymptomatic despite having a chronic disease is often thought of as being healthy and not needing care, and thus can affect uptake of and retention in health services. A study in Uganda for example showed that children were often only tested after becoming visibly ill; 72% of children in the Uganda study presented with advanced HIV disease at their initial visit [8]. Other chronic diseases, such as hypertension and diabetes, are conditions with a high disease burden without symptoms until complications occur [21, 22]. While there may be resistance to preventative care, the experience of caregivers witnessing improvements in their children’s health once in treatment was an important facilitator to remaining in care. Positive messages in awareness campaigns and strengthening information focusing on chronic disease management could help to get and retain parents in care for themselves and their children. Messages should be refined to emphasize the benefits of early infant diagnosis and treatment, including “success stories” from caregiver-child pairs in local communities and messages of hope.

While less frequently reported than individual factors, there were some notable interpersonal and community-related barriers and motivators. Fear of disclosure of the child’s HIV status to family and community members was found to be an influential barrier and is one that has been reported in other studies [9, 10, 23].

The results show that generational conflict in Southern Mozambique remains an important factor. In this regions’ culture, it is common that grandmothers, in the absence of a husband (usually working as migrant workers) decide on health or other issues of their daughters-in-law and grandchildren. This creates conflicts between mother-in-law and daughter-in-law in which young women may not share information with their mothers-in-law. This can prevent mothers-in-law from offering their support, which in turn may lead to daughters not attending or returning to facilities. In an ethnographic study conducted in Mozambique, mothers-in-law have even expelled their daughter-in-laws when discovering that she is taking antiretroviral treatment, which is seen by mothers-in-law as a sign of impurity and that may impede fertility [24]. Mothers-in-law consider that securing childbearing is more important than women’s health, which includes taking antiretroviral drugs. These decision-makers at the intrapersonal level should be included when implementing programs to improve uptake of PMTCT services and targeted with the community messaging mentioned above.

At the institutional level, the lack of human resources is still one of the big challenges in health service delivery in general, which results in prioritizing acute health issues over chronic disease care. Results from our study suggest that making changes at an institutional level, for example reorganizing of patient flow, integrating HIV services into mother and child health services, and task shifting to non-physician health care workers and lay workers could contribute to improved access to care and reducing stigma. One study in Mozambique found increased retention in early infant diagnosis services using direct linkage between maternity and early infant diagnosis services to improve service integration [25] but no effect was seen in another study after an intervention of an integrated services model [26]. It is also notable to see the contrasting results of our study between urban Maputo City, where the reinstituting of a specialized clinic for pediatric HIV was preferred, and the rural areas where identifiable HIV services was seen as stigmatizing. This reflects the need for a comprehensive approach that takes account for the local context.

Barriers of transportation, food, and distance to facility were probed in our research, but surprisingly not reported as frequently as other barriers that were described in this study, though the influence of these barriers was highlighted in a study done in another province in Mozambique [27]. While less frequently mentioned overall, these barriers have the potential to significantly impact health-seeking behavior. This is especially true in the northern region, where long distances exist between villages and health facilities, worsened by the current situation in which patients need to return to the clinic multiple times before getting all the tests and care that they need.

Our study findings show that many barriers and motivators of uptake of and retention in HIV prevention, care and treatment services are similar in sub-Saharan countries and confirm their relevance. Barriers reported from caregivers with children attending South African clinics included their own fears, knowledge, and beliefs about HIV and treatment (e.g. equating HIV infection with death), long waiting times, overcrowding, negative staff attitudes, fear of confidentiality breaches, and lack of money for transport and food. These was also described in a recent review where overcoming those barriers is seen to be crucial to impact pediatric HIV [28]. The main facilitator was seeing children’s health and well-being improve on ART [10]. In Zimbabwe, similar factors were described in a qualitative study among caregivers of HIV positive children [29]. Barriers and motivators for uptake of and retention in services for HIV-infected and exposed children were similar to those of adherence to antiretroviral treatment [30, 31], and to health services in general.

Our study has some limitations. Many of the responses by participants related more directly to the barriers to accessing HIV-related care services in general, including for themselves, other adults, or children, rather than specifically about access to care for their children. This may be due to the fact that they feel that the same responses are valid for both adults and children; that HIV-infected parents will or will not access services for their children for the same reasons that they will or will not access services for themselves. Challenges in reaching caregivers that abandoned care and treatment is another limitation. Results may not be generalizable as we conducted this qualitative study in only five districts of Mozambique. Finally, there is the possible social desirability bias, by community leaders for example, as they are expected to set a good example for their communities and thus may be motivated to respond with anticipated “correct” answers.

## Conclusions

Results of this qualitative study reveal a predominance of individual-level factors that influence health-seeking behavior for HIV-exposed and HIV-infected children in Mozambique. No differences in findings were seen between HIV-exposed and HIV-infected children. Barriers such as the practice of seeking traditional medicine, disbelief in test results from all groups except the caregivers in the community and fear of disclosure of the mother’s and potentially of the child’s HIV status within the family were found to be deeply imbedded in all three study provinces. Caregivers were most often motivated to take children to facilities when they were displaying visible symptoms of an illness. Strategies such as strengthening the active tracing of women during pregnancy and mother-child pairs following delivery, and counseling provided during tracing visits are recommended to increase uptake to services for HIV-exposed children. Lastly, more awareness in the community of preventive care for HIV-exposed children (including early pediatric HIV testing) and involving family decision makers in the preventive and curative care processes could help children to receive the services they deserve.
